# Gas Chromatography–Mass Spectrometry-Based Metabolite Profiling for the Assessment of Freshness in Gilthead Sea Bream (*Sparus aurata*)

**DOI:** 10.3390/foods9040464

**Published:** 2020-04-09

**Authors:** Athanasios Mallouchos, Theano Mikrou, Chrysavgi Gardeli

**Affiliations:** Department of Food Science and Human Nutrition, Agricultural University of Athens, Iera Odos 75, 118 55 Athens, Greece; theano.mikrou@gmail.com (T.M.); agardeli@aua.gr (C.G.)

**Keywords:** sea bream, fish, spoilage, metabolomics, multivariate analysis, biomarkers

## Abstract

Gilthead sea bream (*Sparus aurata*) is one of the most important farmed Mediterranean fish species, and there is considerable interest for the development of suitable methods to assess its freshness. In the present work, gas chromatography–mass spectrometry-based metabolomics was employed to monitor the hydrophilic metabolites of sea bream during storage on ice for 19 days. Additionally, the quality changes were evaluated using two conventional methods: sensory evaluation according to European Union’s grading scheme and *K*-value, the most widely used chemical index of fish spoilage. With the application of chemometrics, the fish samples were successfully classified in the freshness categories, and a partial least squares regression model was built to predict *K*-value. A list of differential metabolites were found, which were distinguished according to their evolution profile as potential biomarkers of freshness and spoilage. Therefore, the results support the suitability of the proposed methodology to gain information on seafood quality.

## 1. Introduction

Gilthead sea bream (*Sparus aurata*) is farmed intensively in Greece and accounts for over half of all production in Europe. In 2018, the volume of production reached 61,000 tons, with a value of EUR 276 million. Greece in particular is expected to double its production by 2030 in order to meet the growing demand and maintain its market position globally [1].

Fish quality is objectively the most important characteristic that affects acceptance by the consumer, and it is dependent on a wide range of factors [2]. Freshness (or degree of spoilage) is a decisive factor in assessing fish quality. Its deterioration begins immediately after slaughter and takes place through biochemical, physicochemical, and microbiological mechanisms [3]. Post-mortem changes depend on species, age, diet, slaughter method, processing, and conditions during transportation and storage, such as temperature, which is the most important factor affecting the commercial life of the product [4]. Preservation on ice is the most common method of maintaining fresh fish, which limits microbial growth, the main cause of spoilage.

The European Community has established common marketing standards for fishery products to assess freshness through organoleptic examination [5]. Thus, the fishing industry must grade the products in three freshness categories defined as Extra, A, and B. Fish not classified in any of these grades are considered unacceptable. Although organoleptic examination is still the most satisfactory way of assessing fish freshness, issues of objectivity and convenience can be claimed if compared with instrumental methods.

From an analytical point of view, several methods have been recommended in order to evaluate fish quality, which rely on the determination of chemical, microbiological, and physical parameters [2]. The *K*-value, one of the most widely used chemical indexes to monitor fish quality, is based on the measurement of adenosine triphosphate (ATP) and its degradation products, namely, adenosine diphosphate (ADP), adenosine monophosphate (AMP), inosine phosphate (IMP), inosine (INO), and hypoxanthine (Hx) [6]. However, the *K*-value is subject to large inter- and intra-species variations and is dependent on many factors [7].

The term “metabolomics” refers to the systematic study of low molecular mass metabolites, which vary under a given set of conditions in the cell, tissue, or organism [8]. In recent years, the metabolomics studies on seafood products have been steadily increasing and have focused mainly on the nutritional status of fish [9], differentiation between wild and farmed fish [10,11,12], and classification according to aquaculture system [13,14,15], but also some steps have been taken towards seafood freshness. More specifically, the changes in metabolic profiles during cold storage have been investigated on yellowtail [16], bogue [17], mussels [18], and salmon [19]. With regards to sea bream, Picone et al. [20] investigated the molecular profiles using ^1^H-NMR only at the beginning and the end of iced storage of fish produced with different aquaculture systems. Heude and co-workers [21] proposed a method based on NMR spectroscopy for the rapid determination of *K*-value and trimethylamine nitrogen content on sea bream, among other fish.

In the present study, gas chromatography–mass spectrometry (GC–MS) was used to monitor the changes in the polar metabolite fraction of sea bream during storage on ice, in order to identify potential markers of freshness and spoilage. Multivariate data analysis was applied to classify fish samples in freshness categories according to EU sensory scheme, and a partial least squares regression (PLS-R) model was built to predict *K*-value.

## 2. Materials and Methods

### 2.1. Fish Provision, Storage, and Sampling

Gilthead sea bream samples (400–600 g, 25–30 cm) were obtained directly from a Greek fish processing plant (PLAGTON S.A., Mitikas, Aitoloakarnania, Greece). Fish were farmed in cages in the geographical area designated as Food and Agriculture Organization (FAO) 37.2.2 (Ionian Sea) and were slaughtered by immersion in ice cold water (hypothermia), packed with flaked ice into self-draining polystyrene boxes, and delivered to the laboratory within 3–4 h of harvesting. Two fish batches, each consisting of 30 ungutted whole fish, were used in the course of two independent storage trials. The fish batches were harvested in April and July of the same year. The fish samples were stored in a refrigerator, and fresh ice was added daily. The harvesting day was considered as day 0 of storage period. The sampling began the next day of storage (day 1), and afterwards continued every 2 days for a total period of 19 days. At each sampling point, three randomly chosen fish were removed from the batch and used for the subsequent analyses.

### 2.2. Sample Preparation for GC–MS Metabolomics

Fish were treated as described in Association of Official Agricultural Chemists (AOAC) Official Method 937.07. The heads, scales, tails, fins, guts, and inedible bones were removed and discarded. Then, fish were filleted to obtain all flesh and skin from head to tail and from top of back to belly on left side only. Each fillet (white muscle with skin) was cut quickly in small cubes and snap-frozen in liquid nitrogen to quench the metabolism. Tissue grinding was performed in a pre-cooled A11 analytical mill (IKA, Wilmington, NC, USA) to obtain a fine frozen powder. The mill was operated in pulse mode for 10–15 s per grinding batch in order to prevent the thawing of the sample. Aliquots (50 mg) of each powdered sample were accurately weighed (± 0.1 mg) into 2 mL Eppendorf tubes with O-ring screw caps (Sarstedt, Germany) and transferred to −80 °C for storage. The remaining quantity of each sample powder was stored at −80 °C in sealed bags and used for the determination of *K*-value. Furthermore, from each sampling point, a suitable quantity (10 g) of fish powder was pooled to obtain a single quality control (QC) sample, which was further processed similarly to unknown samples, as described below.

Tissue disruption and subsequent metabolite extraction was undertaken using a Tissuelyser LT (Qiagen, Germantown, MD, USA) according to a modified Bligh and Dyer method [22]. Pre-chilled and degassed homogenization solvent (525 μL methanol/water, 2:0.625 v/v, HPLC grade), internal standard (50 μL glycine-d5, 0.2 mg/mL in 0.1 M HCl), and two stainless-steel balls (2.5 mm diameter) were added to each Eppendorf tube and, subsequently, the fish powder was homogenized for 2 min at 20 Hz. Then, 200 μL of chloroform was added, and the homogenization was repeated for 1 min. Then, another 200 μL of chloroform was added to each tube, and the contents were mixed for 10 min using a cell shaker. During this process, the samples were always kept on ice. Finally, 200 μL HPLC-grade water was added, and the samples were vortex mixed for 15 s. To initiate phase separation, the samples were centrifuged for 2 min at 12,000 rpm. A total of 100 μL of the aqueous fraction was transferred in new Eppendorf tubes with pre-punctured screw caps and lyophilized overnight (12 h). After replacing the caps with new ones, the sample pellets were stored at -80 °C until required for analysis.

Prior to GC–MS analysis, a two-stage chemical derivatization process was carried out to impart volatility to non-volatile metabolites, while also enabling thermal stability [23,24]. The lyophilized samples were left to reach room temperature for 15 min and then 40 μL of 20 mg/mL methoxyamine solution in pyridine (Acros Organics, Geel, Belgium) was added, and they were then incubated at 30 °C for 90 min in an orbital heating block. Subsequently, 70 μL MSTFA (*N*-methyl-*N*-trimethylsilyltrifluoroacetamide—Acros Organics) was added, and the samples were incubated at 37 °C for 90 min. After cooling the samples for 5 min, 20 μL of retention index solution (*n*-alkanes C10–C24, 0.6 mg/mL in pyridine—Sigma Aldrich, Darmstadt, Germany) was added and the contents were transferred to 200 μL conical insert placed in 2 mL vial with screw cap for further GC–MS analysis.

### 2.3. GC–MS Analysis

GC–MS analysis was carried out using a Shimadzu GCMS QP-2010 Ultra operated with the accompanied GCMS Solution software. Helium was used as a carrier gas at a constant linear velocity of 36 cm/s. Sample injections (1 μL) were performed with AOC 20 s autosampler in split mode (split ratio 1/25). The temperature of the injection port, interface, and ion source was set at 250, 290, and 230 °C, respectively. Separation of compounds was carried out in a MEGA-5HT fused silica capillary column (30 m × 0.25 mm, 0.25 μm film thickness, MEGA S.r.l., Legnano, Italy). Oven temperature was maintained initially at 60 °C for 1 min, then programmed at 10 °C/min to 325 °C, and held for 5 min. The mass spectrometer was operated in electron ionization mode with the electron energy set at 70 eV and a scan range of 70–600 m/z. The samples (QC and blanks included) were analyzed in a predetermined order [24].

### 2.4. Data Processing Procedure

Raw data were processed with MS-DIAL software, which is freely available at the PRIMe website (http://prime.psc.riken.jp/) [25]. Metabolite identification was performed according to the Metabolomics Standards Initiative at four levels [26]:

- MSI level 1 (identified compounds): based on similarity of retention index (RI) and mass spectrum relative to an authentic compound analyzed under identical experimental conditions.
- MSI level 2 (putatively annotated compounds): agreement of retention index (ΔRI < 20) and mass spectrum (match > 850) coming from the publicly available libraries at PRIMe. Amdis (v. 2.72) and NIST MS Search software (v. 2.2) including NIST 14 mass spectral library were used complimentarily.
- MSI level 3 (putatively characterized compound classes): agreement of RI or mass spectrum to known compounds of a chemical class.
- MSI level 4 (unknown compounds).

The resulting output from this procedure was a retention index vs. sample data matrix with related metabolite IDs and peak heights linked to each sample injection. Subsequently, manual data curation was performed, which included the removal of metabolic features detected in < 50% of QC samples; the combination of metabolite rows that had two or more identical peaks, such as sugars; and normalization to sample mass used in extraction. Finally, the data were normalized to the QC samples using a low-order nonlinear locally estimated smoothing function (LOESS) [24], in order to correct for the signal drift within and between analytical blocks. Afterwards, metabolites with relative standard deviation (RSD) > 30% within pooled QCs were removed. The final data matrix was further processed statistically in MetaboAnalyst 4.0 web-based tool suite [27]. This included multivariate and univariate testing as detailed in the Results and Discussion section. Before statistical processing, the data were log-transformed and mean centered. Partial least squares regression (PLS-R) was performed using The Unscrambler X ver. 10.4 (CAMO Software AS, Oslo, Norway).

### 2.5. Freshness Assessment

The freshness rating of raw fish was performed by a panel of three trained assessors according to the European Union’s grading system [5]. This system distinguishes between three freshness categories (Extra, A, B) corresponding to various levels of spoilage. Category E corresponds to the highest quality level, followed by categories A and B, whereas fish graded below B is considered unacceptable for consumption. In order to rate this evaluation, a 0–3 score scale was used (rating of categories: Extra ≥ 2.7, 2 ≤ A < 2.7, 1 ≤ B < 2, unacceptable < 1) according to [28].

### 2.6. ATP Breakdown Products

ATP and its degradation products (ADP, AMP, IMP, Ino, and Hx) were isolated from fish tissue according to Ryder [29]. Chromatography was performed using a JASCO HPLC system (JASCO International Co., Ltd., Tokyo, Japan) consisting of a quaternary pump (PU-2089 Plus), an autosampler (AS-1555), and a photodiode array detector (MD-910). The separation was accomplished with a Luna C18 column (250 mm × 4.6 mm i.d., 5 μm; Phenomenex, Torrance, CA, USA) using gradient elution. Mobile phase A was a 0.05 M phosphate buffer (pH 7) and mobile phase B was acetonitrile (Sigma Aldrich, Louis, MI, USA). The elution program was as follows: 0 min, 100% A; 9 min, 97% A; 15 min, 85% A; 17 min, 60% A. Final conditions were kept for 7 min and the column was equilibrated for 15 min at initial conditions. The flow rate was set at 1 mL/min and the injection volume was 20 μL. The monitoring wavelength was set at 254 nm and the molar concentration of ATP breakdown products were calculated from their corresponding calibration curves using the external standard method. The *K*-value (%) was calculated from Equation (1):

(1) $$K\left(\%\right)=\frac{\left(Ino+Hx\right)}{\left(ATP+ADP+AMP+IMP+Ino+Hx\right)}\times 100\%$$

## 3. Results and Discussion

### 3.1. Freshness Assessment Using Classical Methods

**Figure 1:**
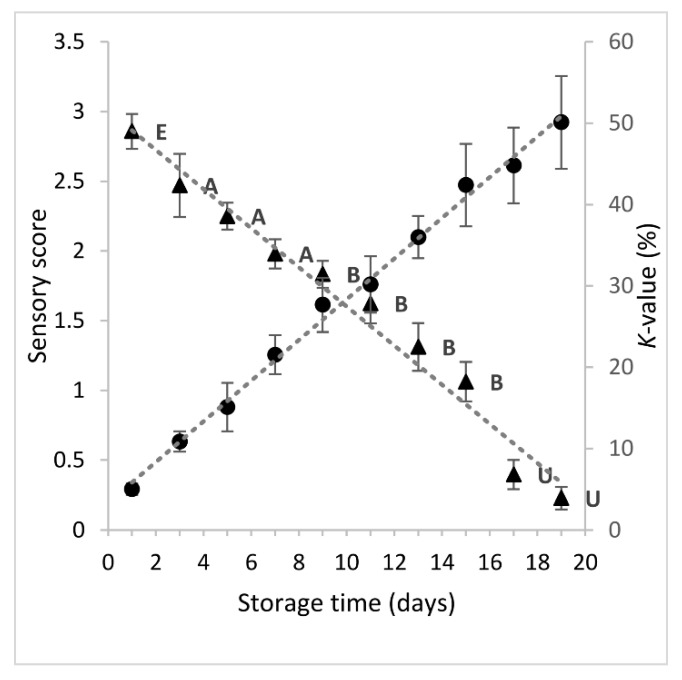
Changes in sensory score (-▲-) and *K*-value (-●-) of sea bream stored in ice. Each point represent the mean value of six replicate measurements (three fish samples x two storage experiments at each sampling point). Error bars denote standard deviation. The labels of sensory scores denote the freshness category according to EU grading system.

Quality deterioration of fish during storage on ice was monitored using a sensory method (EU grading system), and a chemical one (*K*-value), which is based on the measurement of ATP breakdown products. The changes in sensory score and *K*-value of sea bream during 19 storage days on ice are shown in Figure 1. As expected, the sensory score decreased linearly (*y* = −0.1404*x* + 3.0057), showing high negative correlation with storage time (*r* = -0.9880, *p* < 0.001). Until day 1 of storage, the freshness rating of fish was evaluated as Extra. From day 3 to day 7, the freshness of fish was rated A, whereas the category B was assigned to fish stored between 9–15 days. The limit of acceptability of raw sea bream stored on ice was about 16–17 days. As the sensory quality of fish decreased, the *K*-value increased linearly (*y* = 2.5033*x* + 3.3305), showing a high positive correlation with storage time (*r* = 0.9974, *p* < 0.001) and a negative correlation with sensory score (*r* = −0.9809, *p* < 0.001). When the fish was considered unacceptable (day 17), the *K*-value was 45%. Similar findings have been reported by others authors [6,30,31]. Small variations could be attributed to the different rearing area and farming method among others.

### 3.2. Freshness Assessment Using GC–MS Metabolomics

Before starting the real data elaboration, method performance was evaluated using several quality control criteria. The primary requirement was to check the relative abundance of amino acids and sugar trimethylsilyl (TMS) derivatives in QCs. A detailed description is provided elsewhere [23]. After passing the above criteria, the internal standard performance was checked. Deuterated glycine-d5 was added in every sample (including QCs and blanks) to monitor the extraction procedure. For this reason, the RSD% of peak height was calculated and the value obtained was 5.4% for QCs (*n* = 15) and 15.3% for samples (*n* = 54). As a last check, a preliminary principal component analysis (PCA) was obtained with the peak heights of the entire dataset. A tight clustering of the QCs, as well as blank samples, was observed in the score plot (Figure S1), which is a further confirmation of the robustness of the analytical procedure, not only for the internal standard but for the whole fingerprint of fish.

**Figure 2:**
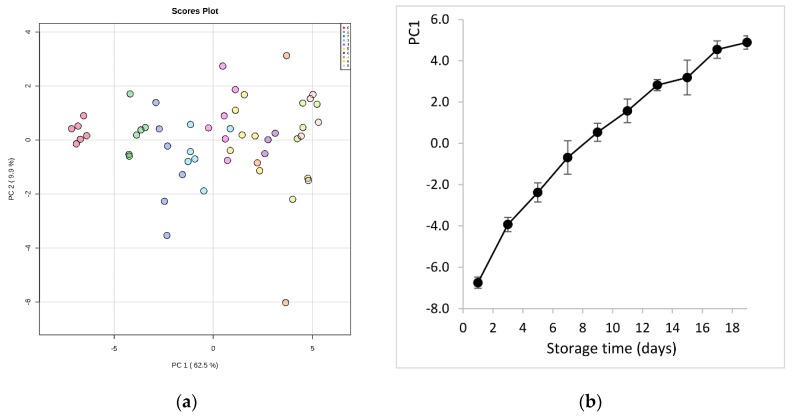
(**a**) Principal component analysis (PCA) score plot derived from the hydrophilic metabolites of sea bream during storage on ice. The legend indicates the sampling sequence (0-1-2-3-4-5-6-7-8-9) that corresponds to storage day (1-3-5-7-9-11-13-15-17-19), respectively; (**b**) evolution of sea bream spoilage as described by PC1 scores values vs. storage time on ice.

After data curation, the samples were grouped in 10 classes (from 0 to 9) according to the sampling sequence during fish storage on ice (class 0 represents the first day of storage, class 1 the third day, etc.). Principal component analysis (PCA) of data exhibited a significant ability to separate samples according to storage time (Figure 2a). It is evident that very fresh samples (class 0, day 1) were clearly separated from the other classes, and most distinctively from those at the later stages of storage (class 8, 9). This shows that there is quantitative information in these data, as PC1, with an explained variance of 62.5%, is the component that describes the evolution of fish spoilage. This was depicted even more clearly in the plot of PC1 scores values vs. storage time (Figure 2b). Thus, PC1 can condensate all information of the metabolite features and give a measure of the molecular quality of fish.

**Figure 3:**
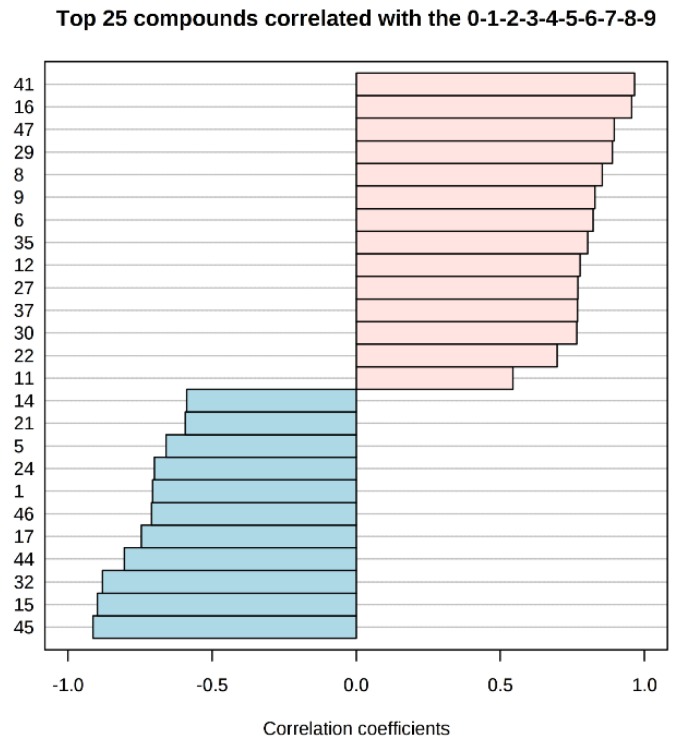
Pattern recognition—Spearman’s correlation analysis showing the top 25 metabolite features correlated significantly with sampling sequence (0-1-2-3-4-5-6-7-8-9 is equivalent to storage day 1-3-5-7-9-11-13-15-17-19). Each row represents the most significant metabolite identified from the test (*p* < 0.05). The *x*-axis shows correlation score, whereas the *y*-axis corresponds to gas chromatography–mass spectrometry (GC–MS) peak number from peak index (see Table 1).

Studying the multivariate loadings values revealed various metabolites that either increased or decreased with fish storage. The most important loadings were established and confirmed by Kruskal–Wallis ANOVA, as well as by performing Spearman’s correlation analysis. Figure 3 shows the top 25 highly correlating and significant metabolites (*p* < 0.05) that either increased (shown in red; positive *R*) or decreased during storage (shown in blue; negative *R*).

**Figure 4:**
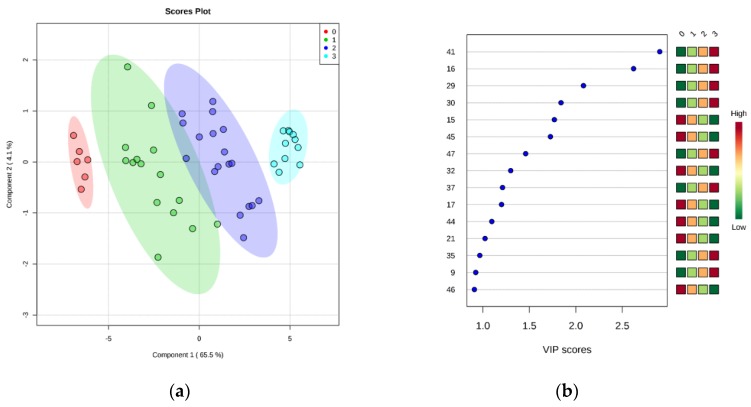
(**a**) Partial least squares discriminant analysis (PLS-DA) scores plotted for freshness grades of sea bream stored on ice. The legend indicates the four EU grades: Extra (0), A (1), B (2), unacceptable (3). (**b**) Top 15 metabolite features based on variable importance in projection (VIP) scores from PLS-DA. The *x*-axis shows the scores whereas the *y*-axis corresponds to GC–MS peak number from peak index (see Table 1). Color bars show median intensity of metabolite feature in the respective group.

After these encouraging results, the data were grouped into four classes (0 to 3) representing the EU freshness grades (i.e., grade Extra: class 0; grade A: class 1; grade B: class 2; unacceptable: class 3), as described in EC no. 2396/1996. Partial least squares discriminant analysis (PLS-DA) was carried out in order to find a discriminant index of freshness. It is evident that the supervised model (Figure 4a) can clearly classify the samples into the correct freshness grade. Although there was some overlap of the confidence ellipses of grade A (class 1) and B (class 2), the grade Extra (class 0) was further apart from unacceptable samples (class 3). Similarly to the aforementioned PCA results, it seems that this separation was described mainly by PC1, which accounted for the 65.5% of model variance. The optimal number of components, as calculated by 10-fold cross-validation, was 3 (Figure S2). The predictive ability of the model (*Q*^2^), accuracy, and coefficient of determination (*R*^2^) were satisfactory (0.94, 0.97, and 0.76, respectively). The significance of class discrimination was verified by performing a permutation test (*p* < 0.001; 0/1000), and the performance was measured using group separation distance (B/W ratio) [32] (Figure S3). The variable importance in projection (VIP) scores were also calculated from the PLS-DA, and Figure 4b highlights the top 15 highly significant metabolites that were identified for each freshness grade.

The combined result of PCA, PLS-DA, and Kruskal–Wallis ANOVA is summarized in Table 1, which shows a list of metabolites significantly correlated with fish storage on ice. They can be distinguished in two groups—in the first group were metabolites whose relative content increased significantly during storage, whereas the second group comprised metabolites with a decreasing trend. Thus, we can infer that the first group of compounds constitute potential markers of spoilage, whereas the second group could be markers of freshness. Their respective evolution pattern during storage is summarized in Tables S1 and S2. The relative concentration of six amino acids (leucine, isoleucine, valine, phenylalanine, tyrosine, methionine) increased during storage on ice as a result of autolysis and bacterial spoilage. Similar findings were observed in bogue [17] and salmon [19]. On the contrary, the observed decrease of glycine and glutamic acid probably indicates that their degradation by microorganisms occurred in a higher rate than their release from muscle proteins. This is in contrast to the aforementioned studies, but can be rationalized by the different spoilage microorganisms developing in each fish species. The amount of inosine increased during storage, as expected, and was confirmed also by HPLC analysis of ATP breakdown products. The levels of succinic, malic, and fumaric acid, involved in the Krebs cycle, decreased during storage, thus indicating a preferential consumption for bacterial growth. In fact, organic acids or amino acids rather than glucose are the preferred carbon sources for *Pseudomonas* [33], the dominating spoilage genus in sea bream at low temperatures [34]. The increasing trend of some sugars, such as ribose and galactose, has been observed previously in other aquatic products, such as mussels and yellowtail fish [16,18]. On the contrary, the sugar phosphates, such as ribulose 5-phosphate, which is the end product of pentose phosphate pathway (oxidative branch), decreased significantly with storage, and thus may represent freshness markers. It should be noted here that the interpretation of the biological significance of metabolomics data is not always straightforward. The main difficulty arises from the nature of freshness loss, which is a process described primarily by two different phenomena—the autolysis from endogenous enzymes and the spoilage due to microbial growth. In addition, the whole picture is complicated by the evolution of microbial diversity that leads to shifts of metabolite profiles.

**Figure 5:**
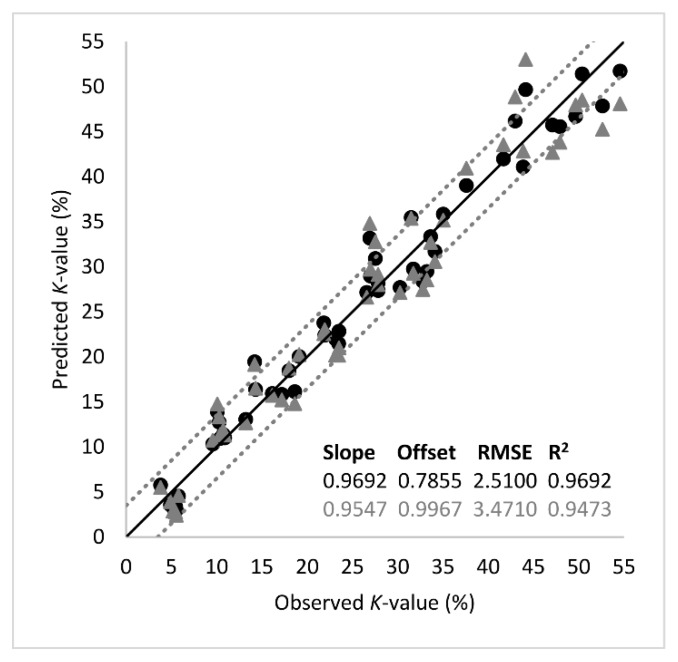
Comparison between the observed and predicted *K*-values (%) by the partial least squares regression (PLS-R) model based on important metabolites listed in Table 1. The shape of symbols denotes the calibration (-●-) and the validation (-▲-) set (black line: the ideal *y* = *x* line; dotted lines: ± 3.5% *K*-value).

The analytes of Table 1 were used further in PLS-R as input variables (predictors, *X*) in order to predict *K*-value (output variable, *Y*). Segmented cross validation was employed using 18 segments with three replicate samples each. Thus, each segment corresponded to a sampling point (18 segments = 9 sampling points × 2 fish batches). External validation using an independent test set was not performed due to the relatively small number of samples (*n* = 54) under study. The optimal number of factors was 3 and explained the 95% of total variance. The performance metrics and the regression line of the model are presented in Figure 5. The root mean square error (RMSE) and the coefficient of determination (*R*^2^) at the validation stage suggested good prediction performance, with their values being 3.4710 (*K*-value %) and 0.9473, respectively. According to the slope of the regression line (0.9546), there was an almost perfect linear relationship between the predicted and the measured *K*-values.

## 4. Conclusions

The present study demonstrated for the first time that GC–MS-based metabolomics is an efficient tool to monitor the quality loss of sea bream during storage on ice. We have clearly presented a panel of hydrophilic metabolites linked directly to storage time of fish that could be used as potential markers of freshness and spoilage. Additionally, with the application of multivariate data analysis, the samples were successfully classified to freshness grades, and the *K*-value was predicted using a PLS-R model. Therefore, our results support the suitability of the proposed methodology to gain information on seafood quality. However, we should note that an approach based on the hydrophilic fraction of metabolites solely, as described here, is not enough for the complete elucidation of the post-mortem changes occurring at the molecular level. Hence, future applications should include the investigation of the lipophilic metabolites using larger-scale storage experiments in combination with microbiological and sensorial data.

## Figures and Tables

**Table 1:** Metabolites that either increased or decreased significantly^1^ during storage of sea bream on ice.

Peak Number	Significant Metabolites	MSI Level	Identifier from Relevant Database
	*Increasing trend*		
41	Gluconic acid	2	HMDB0000625
16	Glyceric acid	2	CHEBI:32398
29	Ribose	2	CHEBI:47014
37	Galactose	1	CHEBI:4139
8	Ethanolamine	2	CHEBI:16000
47	Inosine	2	CHEBI:17596
9	Leucine	1	CHEBI:25017
6	Valine	1	CHEBI:16414
27	Phenylalanine	1	CHEBI:17295
35	Tyrosine	2	CHEBI:17895
12	Isoleucine	2	CHEBI:17191
22	Methionine	1	CHEBI:16643
11	Glycerol	1	CHEBI:17754
30	Ribitol	2	CHEBI:15963
	*Decreasing trend*		
45	Ribulose 5-phosphate	2	CHEBI:17363
44	Arabinose-5-phosphate	2	CHEBI:16241
46	Sugar phosphate_2381^2^	3	N/A
32	Glycerol 3-phosphate	2	CHEBI:15978
15	Succinic acid	1	CHEBI:15741
17	Fumaric acid	1	CHEBI:18012
21	Malic acid	1	HMDB0000744
2	Lactic acid	1	CHEBI:78320
5	a-Aminobutyric acid	2	CHEBI:35621
24	Creatinine	2	CHEBI:16737
1	Methylamine	2	CHEBI:16830
14	Glycine	1	CHEBI:15428
26	Glutamic acid	2	HMDB00148

^1^ According to the combined results of Kruskal–Wallis ANOVA, Spearman’s correlation analysis, and VIP scores from PLS-DA. ^2^ The number denotes the *n*-alkane retention index in MEGA HT-5 column.

## Supplementary Materials

The following are available online at https://www.mdpi.com/2304-8158/9/4/464/s1, Figure S1: PCA score plot for the entire data set before data curation. The legend indicates the type of samples (BL: blank, QC: quality control, S: sample). Figure S2: PLS-DA classification using different number of components. The red star indicates the best classifier based on Q2. Figure S3: PLS-DA model validation by permutation tests based on group separation distance (B/W). Table S1: Most significant metabolites with an increasing trend during storage of sea bream on ice. The *y*-axis represents the relative amount before and after variable transformation, respectively. The *x*-axis shows sampling sequence that is equivalent to storage time. Table S2: Most significant metabolites with a decreasing trend during storage of sea bream on ice. The *y*-axis represents the relative amount before and after variable transformation, respectively, for the bar plot and the box plot. The *x*-axis shows sampling sequence that is equivalent to storage time.
