# Supplementary material for: Gas Chromatography–Mass Spectrometry-Based Metabolite Profiling for the Assessment of Freshness in Gilthead Sea Bream (Sparus aurata)
Source: Foods. 2020 Apr 9;9(4):464. doi: 10.3390/foods9040464 (PMC7231230; doi:10.3390/foods9040464)
Supplement: Supplementary file 1 [file foods-09-00464-s001.zip › Figure_S1.pdf]

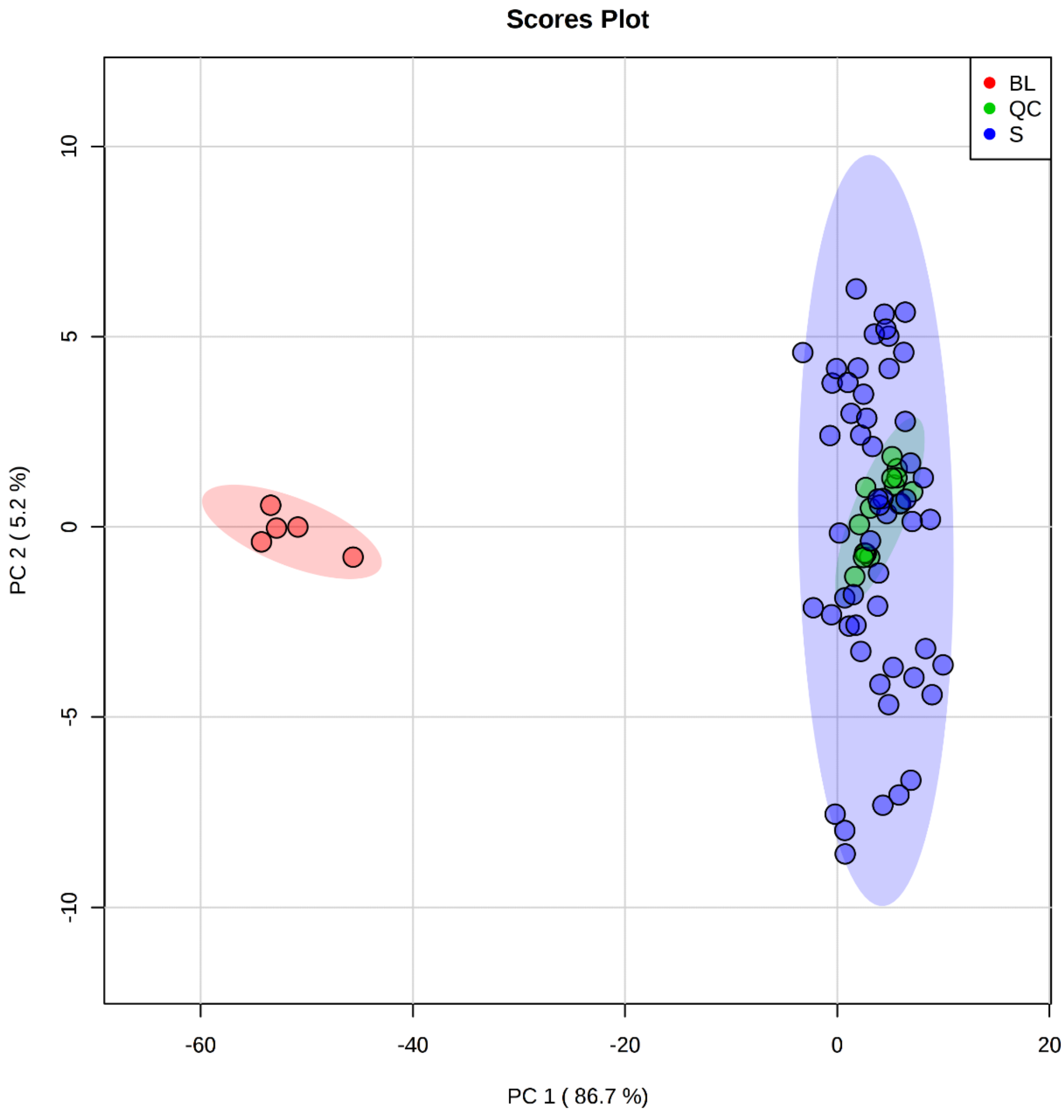

Figure S1: PCA score plot for the entire data set before data curation. The legend indicates the type of samples (BL-blank, QC-quality control, S-sample).
