# Supplementary material for: Gas Chromatography–Mass Spectrometry-Based Metabolite Profiling for the Assessment of Freshness in Gilthead Sea Bream (Sparus aurata)
Source: Foods. 2020 Apr 9;9(4):464. doi: 10.3390/foods9040464 (PMC7231230; doi:10.3390/foods9040464)
Supplement: Supplementary file 1 [file foods-09-00464-s001.zip › Figure_S2.pdf]

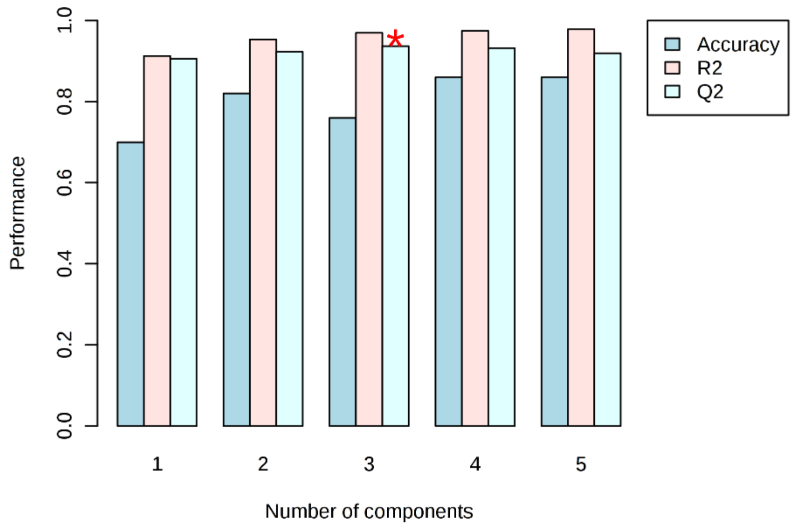

Figure S2: PLS-DA classification using different number of components. The red star indicates the best classifier based on Q2.
