# Supplementary material for: Gas Chromatography–Mass Spectrometry-Based Metabolite Profiling for the Assessment of Freshness in Gilthead Sea Bream (Sparus aurata)
Source: Foods. 2020 Apr 9;9(4):464. doi: 10.3390/foods9040464 (PMC7231230; doi:10.3390/foods9040464)
Supplement: Supplementary file 1 [file foods-09-00464-s001.zip › Table S1.pdf]

Table S1: Most significant metabolites with an increasing trend during storage of sea bream on ice. The *y*-axis represents the relative amount before and after variable transformation, respectively. The *x*-axis shows sampling sequence which is equivalent to storage time.

Metabolites

41

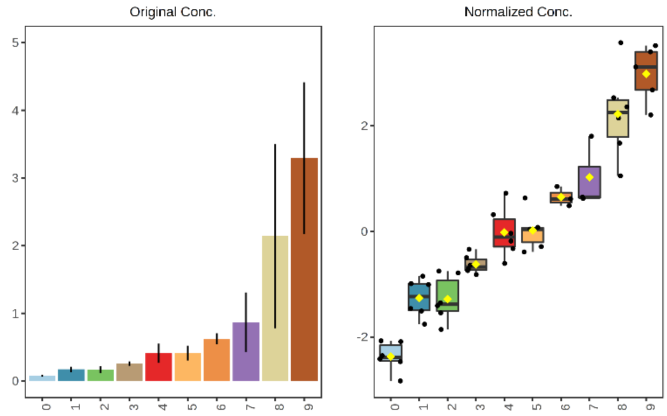

Gluconic acid

16

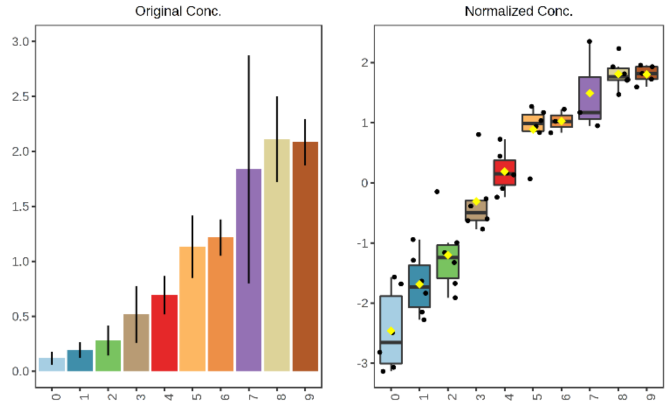

Glyceric acid

29

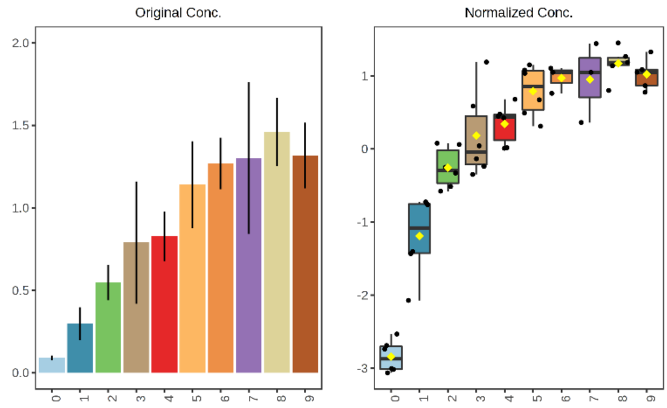

Ribose

37

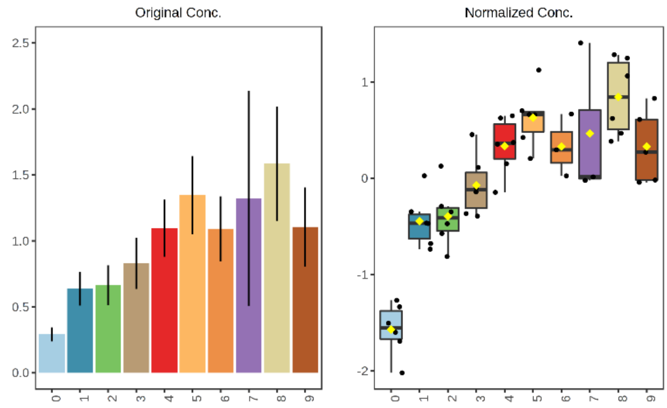

Galactose

8

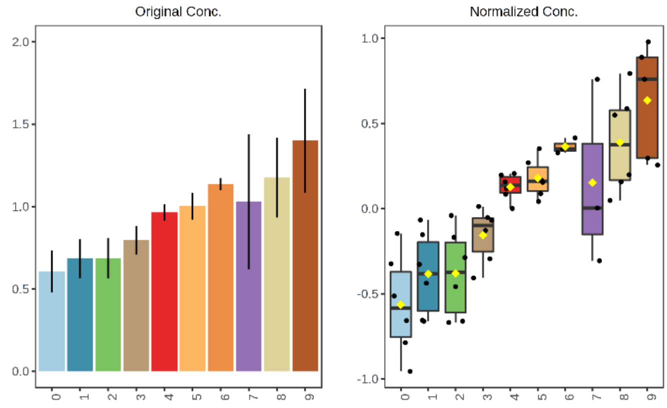

Ethanolamine

47

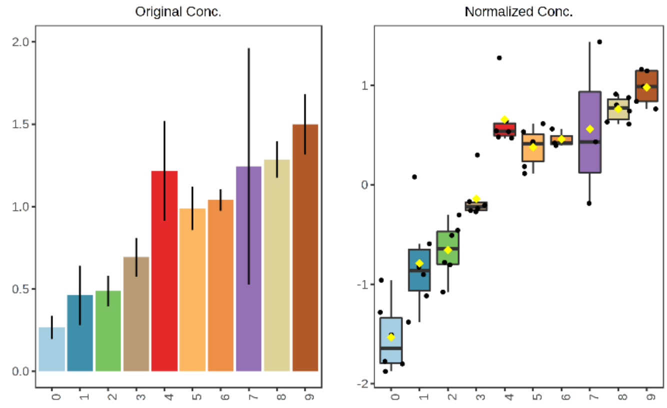

Inosine

9

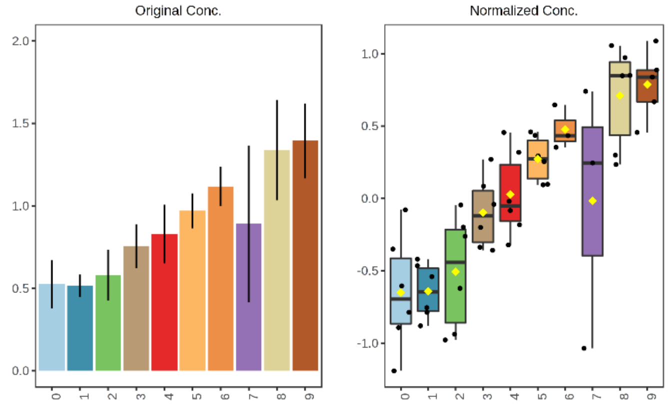

Leucine

6

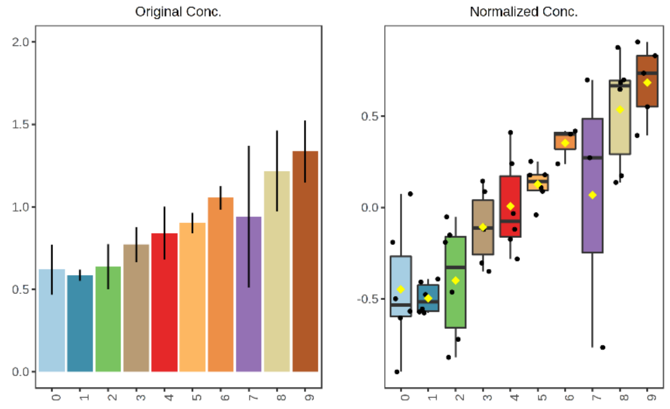

Valine

27

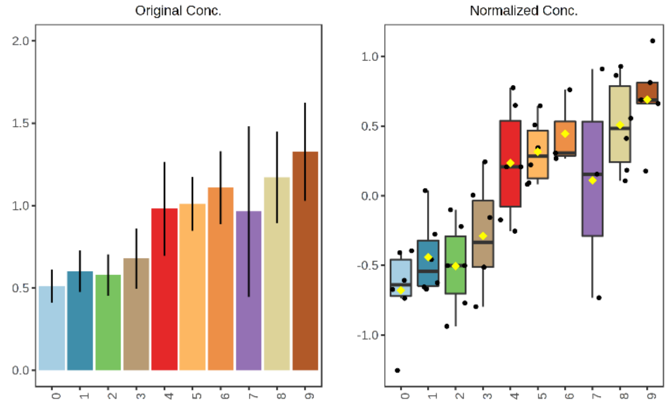

Phenylalanine

35

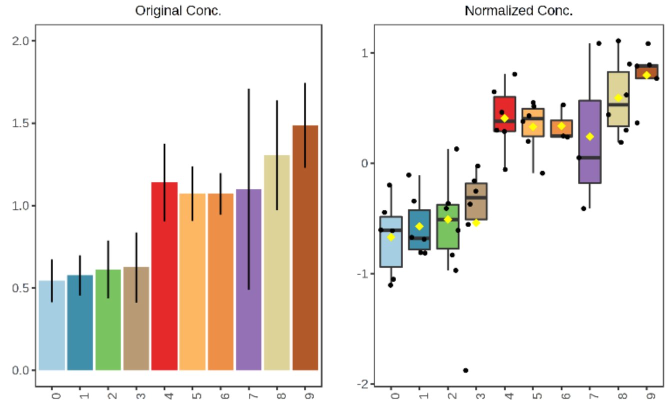

Tyrosine

12

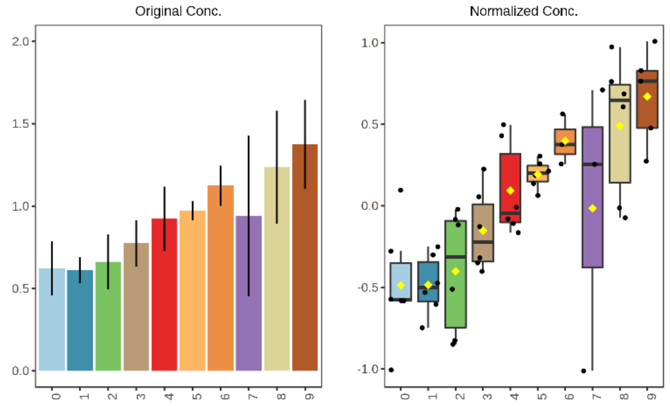

Isoleucine

22

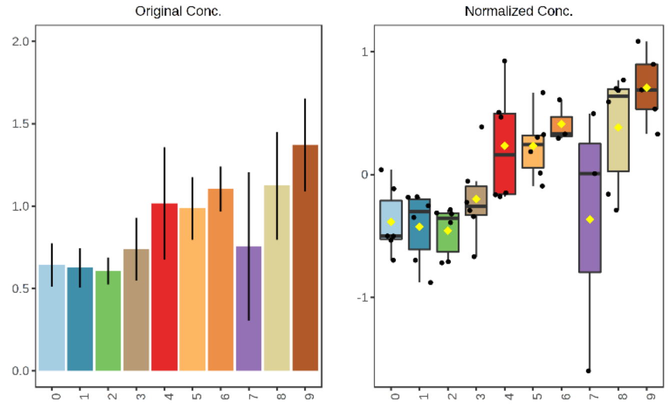

Methionine

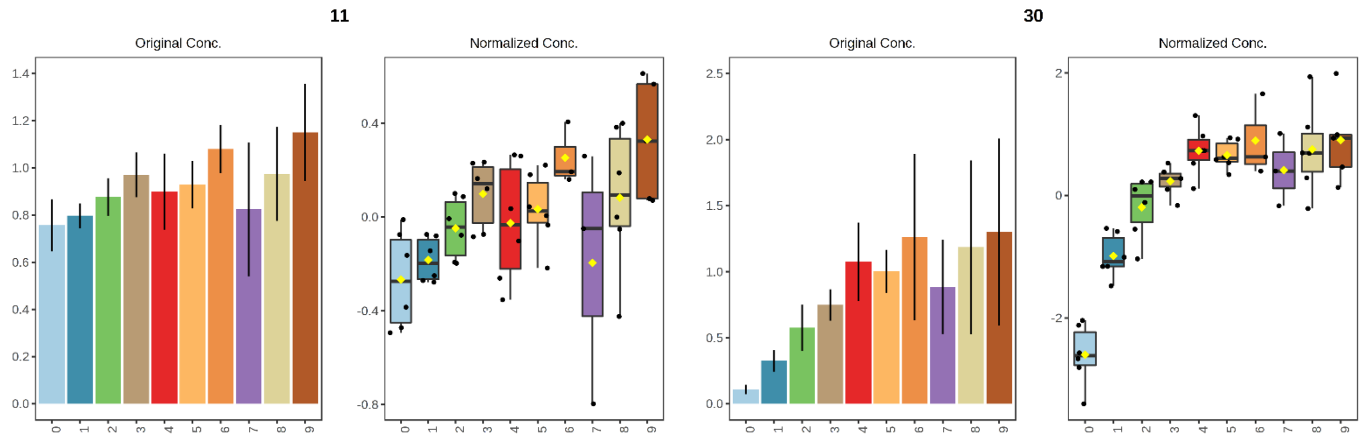

Glycerol

Ribitol
