# Supplementary material for: Gas Chromatography–Mass Spectrometry-Based Metabolite Profiling for the Assessment of Freshness in Gilthead Sea Bream (Sparus aurata)
Source: Foods. 2020 Apr 9;9(4):464. doi: 10.3390/foods9040464 (PMC7231230; doi:10.3390/foods9040464)
Supplement: Supplementary file 1 [file foods-09-00464-s001.zip › Table S2.pdf]

Table S2: Most significant metabolites with a decreasing trend during storage of sea bream on ice. The *y*-axis represents the relative amount before and after variable transformation, respectively for the bar plot and the box plot. The *x*-axis shows sampling sequence which is equivalent to storage time.

Metabolites

45

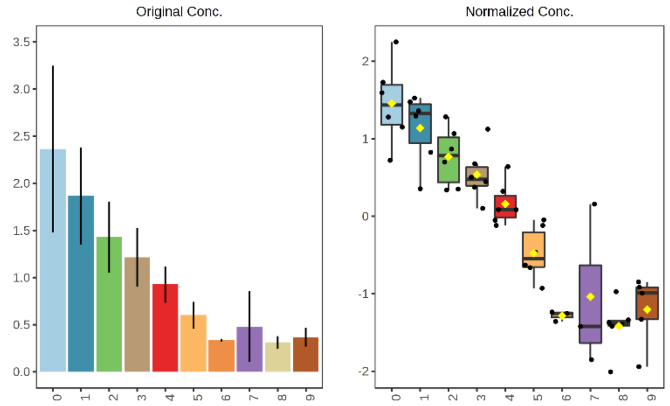

Ribulose 5-phosphate

44

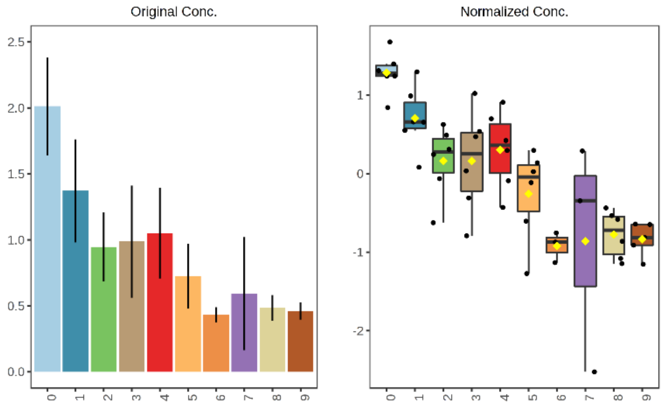

Arabinose-5-phosphate

46

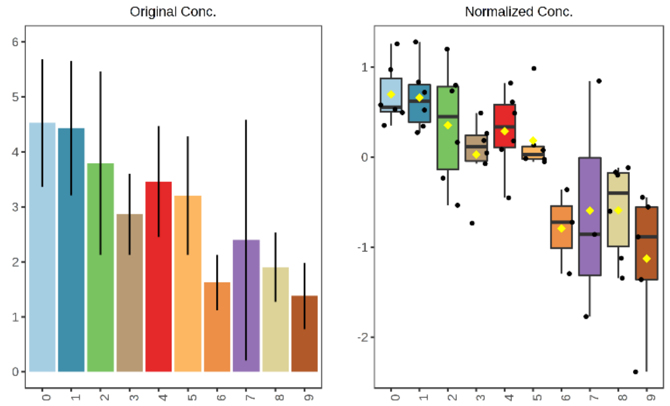

Sugar phosphate_2381

32

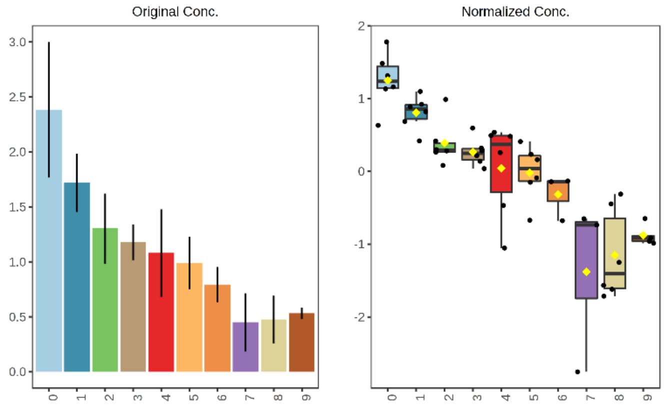

Glycerol 3-phosphate

15

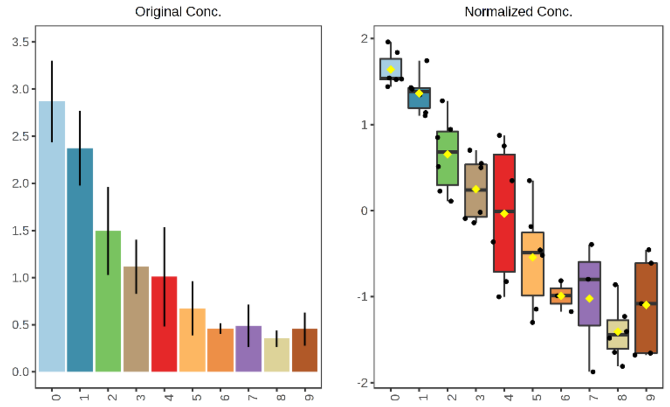

Succinic acid

17

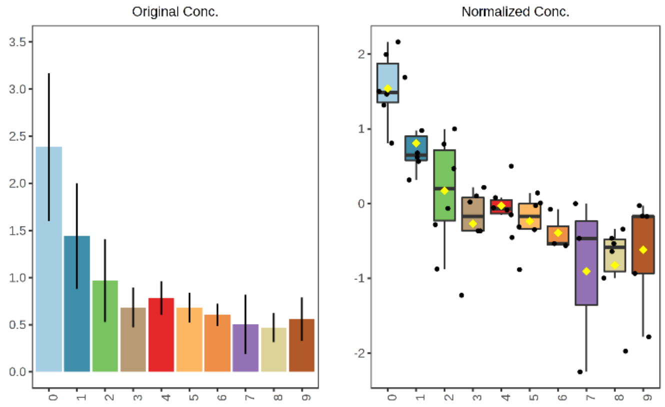

Fumaric acid

21

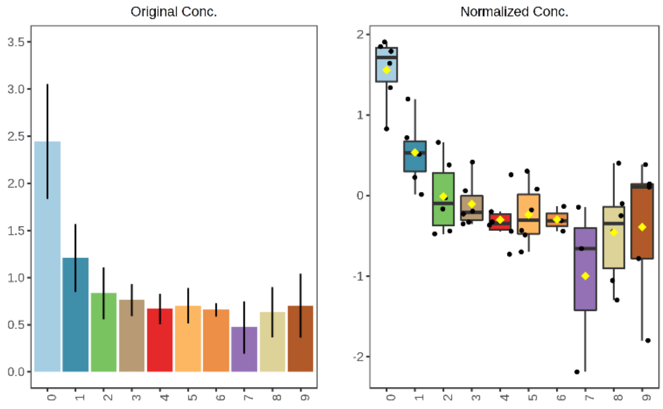

Malic acid

2

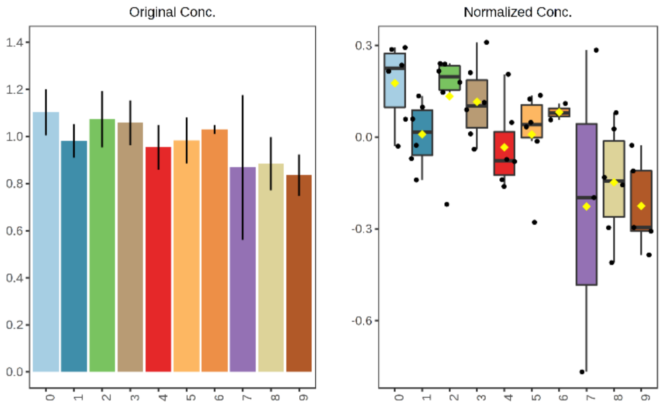

Lactic acid

5

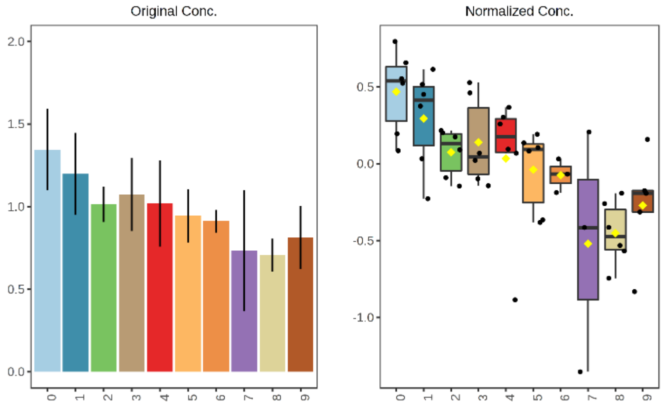

a-Aminobutyric acid

24

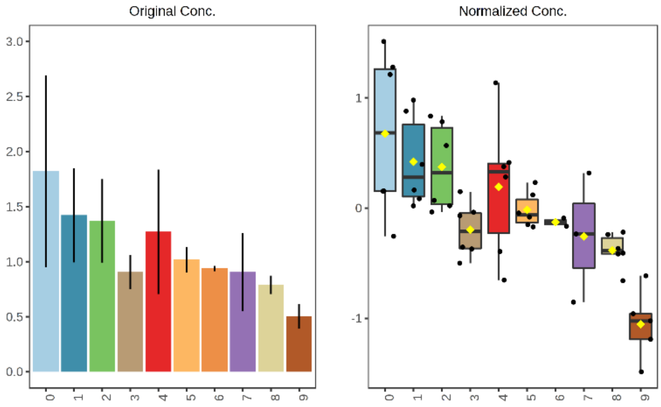

Creatinine

1

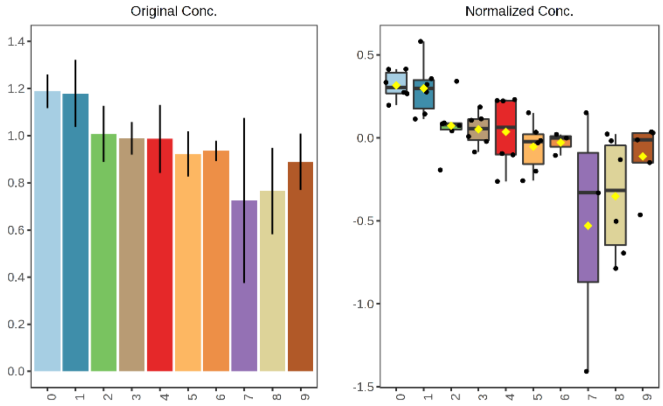

Methylamine

14

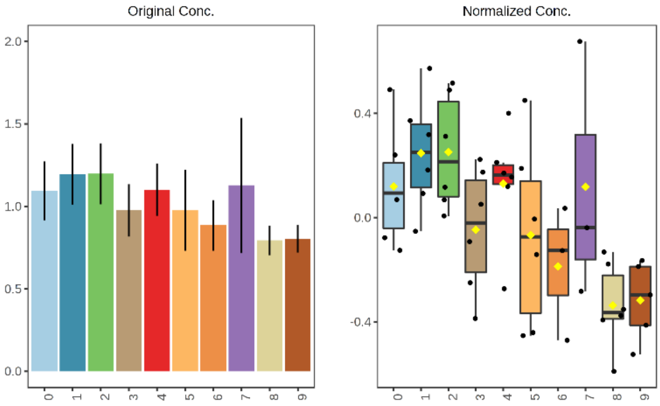

Glycine

26

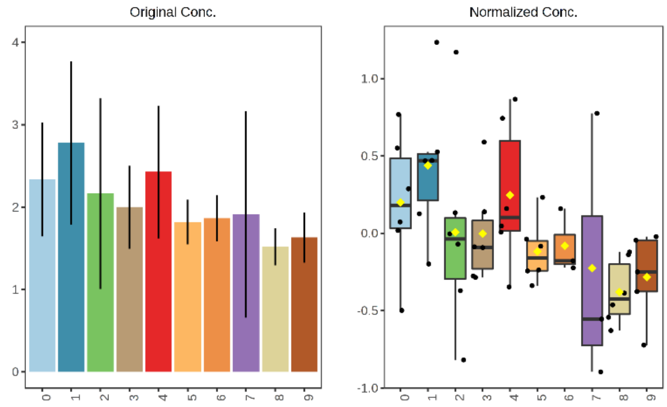

Glutamic acid
